# Colonic Immune Suppression, Barrier Dysfunction, and Dysbiosis by Gastrointestinal *Bacillus anthracis* Infection

**DOI:** 10.1371/journal.pone.0100532

**Published:** 2014-06-19

**Authors:** Yaíma L. Lightfoot, Tao Yang, Bikash Sahay, Mojgan Zadeh, Sam X. Cheng, Gary P. Wang, Jennifer L. Owen, Mansour Mohamadzadeh

**Affiliations:** 1 Department of Infectious Diseases and Pathology, University of Florida, Gainesville, Florida, United States of America; 2 Division of Gastroenterology, Hepatology and Nutrition, Department of Medicine, University of Florida, Gainesville, Florida, United States of America; 3 Division of Gastroenterology, Department of Pediatrics, University of Florida, Gainesville, Florida, United States of America; 4 Division of Infectious Diseases and Global Medicine, Department of Medicine, University of Florida, Gainesville, Florida, United States of America; 5 Department of Physiological Sciences, College of Veterinary Medicine, University of Florida, Gainesville, Florida, United States of America; University of Pittsburgh, United States of America

## Abstract

Gastrointestinal (GI) anthrax results from the ingestion of *Bacillus anthracis*. Herein, we investigated the pathogenesis of GI anthrax in animals orally infected with toxigenic non-encapsulated *B. anthracis* Sterne strain (pXO1^+^ pXO2^−^) spores that resulted in rapid animal death. *B. anthracis* Sterne induced significant breakdown of intestinal barrier function and led to gut dysbiosis, resulting in systemic dissemination of not only *B. anthracis*, but also of commensals. Disease progression significantly correlated with the deterioration of innate and T cell functions. Our studies provide critical immunologic and physiologic insights into the pathogenesis of GI anthrax infection, whereupon cleavage of mitogen-activated protein kinases (MAPKs) in immune cells may play a central role in promoting dysfunctional immune responses against this deadly pathogen.

## Introduction

Gastrointestinal (GI) anthrax, named for its primary route of infection, is an acute infectious disease resulting from the ingestion of the spore-forming, Gram-positive bacterium, *Bacillus anthracis* [1]. Anthrax can also be contracted via inhalation or cutaneous exposure, with inhalation anthrax having the highest mortality rate of the three clinical subtypes [2]. Disease-causing *B. anthracis* spores primarily infect grazing animals, but humans may be exposed to anthrax through the handling of infected animals and animal products, the consumption of tainted meat, or through intentional exposure [1]. Independent of the route of entry, unchecked infection rapidly becomes systemic and death occurs due to septicemia and/or toxemia [3].

Within fully virulent *B. anthracis* strains, two large plasmids, pXO1 and pXO2, are composed of the genes needed for toxin production and capsule formation, respectively, and both plasmids are necessary for complete virulence [4], [5]. The pXO1 encodes protective antigen (PA), lethal factor (LF), and edema factor (EF); lethal toxin (LT) comprises PA+LF, while edema toxin (ET) comprises PA+EF. Via these two toxins, *B. anthracis* evades and inhibits critical signals of the innate and adaptive immune systems [6]. The poorly immunogenic anthrax capsule is encoded on pXO2 and consists of poly-γ-D-glutamic acid, which protects *B. anthracis* from phagocytosis and complement binding [7], [8]. Several therapeutic strategies have targeted specific *B. anthracis* virulence factors [9], [10]; however, development of next generation vaccines and therapeutics against *B. anthracis* requires a better understanding of disease pathogenesis in humans. In particular, insufficient data exist regarding the pathogenesis of GI anthrax [11]–[13]. GI *B. anthracis* infection is not only a persistent and major problem in developing countries, but also poses a threat in biological warfare, whereby intentional contamination of food sources may occur [1].

Here, we report that GI *B. anthracis* spore infection results in swift morbidity and mortality and is associated with pathogen dissemination throughout visceral organs by induction of leakage in the intestinal barrier and significant changes in the gut’s microbial composition, all of which may orchestrate dysfunctional immune responses. A greater understanding of the pathogenesis of GI anthrax and molecular studies of the “microorganism-mammalian immune defense interface” [14] is imperative and may result in improvement of a protective vaccine in man.

## Materials and Methods

### Mice and Ethics Statement

A/J mice were purchased from the Jackson Laboratory and bred in-house in the animal facility at the College of Veterinary Medicine, University of Florida. For microbiota composition experiments, mice were tested after a minimum of two generations of in-house breeding. Mice were used at 6–8 weeks of age in accordance with the Animal Welfare Act and the Public Health Policy on Humane Care. All procedures were approved by the Institutional Animal Case and Use Committee (IACUC) at the University of Florida under protocol number 201107129, and all efforts were made to minimize animal suffering. Infected mice were monitored every 24 hours and were humanely euthanized when signs of advanced infection (e.g., difficulty breathing) were noted; in some cases, mice died as a direct result of the infection before euthanasia could take place. Euthanasia was performed by prolonged inhalation of isoflurane and confirmed by cervical dislocation.

### *B. anthracis* Spore Preparation and Mouse Infections

Spores were prepared with a toxigenic non-encapsulated strain of *B. anthracis* (Sterne), as described previously [15] with the approval of the Institutional Biosafety Committee (IBC) at the University of Florida. To calculate final concentrations, serial dilutions were grown in triplicate on lysogeny broth agar plates and colonies counted. For survival studies, mice were orally infected with 10^5^ spores (n = 10), 10^7^ spores (n = 10), or 10^9^ spores (n = 20) in a final volume of 100 µL with a reusable, 30 mm, 20 gauge, barrel-tipped feeding needle after fasting for 4 hours; infected mice were monitored, and deaths recorded. For immunologic and microbiota composition studies, A/J mice (n = 10/group) were orally infected with Sterne spores (10^9^ spores/100 µL PBS/mouse) for the specified time points. Groups of A/J mice (n = 10/group) were also either orally gavaged or injected intraperitoneally (i.p.) with 125 µg LT (PA+LF) and monitored for morbidity and death.

### Histopathology

Sterne-infected A/J mice were sacrificed at various days post-infection and the colon, spleen, liver, kidney, and lungs surgically excised for analyses. Tissues were fixed, sectioned, and stained with hematoxylin and eosin (H&E) by Histology Tech Services (Gainesville, FL). Histopathology and bacterial dissemination in infected mice were analyzed by a boarded veterinary pathologist (JLO). In some cases, cytocentrifugation of single cell suspensions from spleen, mesenteric lymph nodes (MLNs), and bronchoalveolar lavage (BAL) fluid were evaluated. Neither vegetative bacilli nor spores could be detected in the lungs prior to three days post-infection, confirming that spores were not accidently introduced into the respiratory tract during oral gavage.

### *Ex vivo* Trans Epithelial Electrical Resistance (TEER), Short-circuit Current (I_SC_) and Trans-epithelial Conductance (G_T_) Measurements of Intestinal Tissues

Differences in TEER and electrogenic ion transport in the colons of Sterne-infected versus uninfected mice were quantified by measuring the short circuit current responses of isolated colonic tissues mounted in modified Ussing chambers, as previously described [16]. Briefly, Sterne-infected (day 3) and uninfected A/J mice were euthanized and colons quickly isolated. Segments were cut open along the mesenteric border into a flat sheet and flushed with ice-cold HEPES-Ringer solution; intact, full-thickness segments containing all of the layers of colons were used. The intestinal sheets were mounted between two halves of a modified Ussing chamber (Physiologic Instruments, San Diego, CA) and short-circuited by a voltage clamp (VCC MC6, Physiologic Instruments) with correction for solution resistance. The exposure area was 0.3 cm^2^. The mucosal and serosal surfaces of the tissues were bathed in reservoirs with 3 mL HEPES-Ringer solution containing 100 mM NaCl, 25 mM HCO_3_, 1.5 mM CaCl_2_, 1.5 mM MgCl_2_, 5 mM KCl, 10 mM glucose, and 22 mM HEPES, pH 7.4, maintained at 37°C and continuously bubbled with 95% O_2_ and 5% CO_2_. Tissues and solutions were maintained at 37°C by surrounding water jackets. Tissues were allowed a 30 min stabilization period before measurements were recorded at basal and challenged conditions. The delta current (Δ*I_SC_*) before and after challenge was used to estimate electrogenic current stimulation by external stimuli; the post-challenge secretory current was measured 10 minutes after challenge as basolateral bumetanide-sensitive *I_SC_*. Throughout the experiments, tissues were constantly short-circuited by clamping the transepithelial potential at 0 mV, except for 1 second intervals every 20 seconds when tissues were clamped at±3 mV and *G_T_* determined. Data were acquired via DATAQ instruments (Akron, OH) and processed using the Acquire & Analyze software (Warner Instruments, Hamden, CT).

### Real-time PCR

RNA was isolated from distal colons of mice with Aurum Total RNA Kit (Bio-Rad). iScript Select cDNA Synthesis Kit (Bio-Rad) was used for reverse transcription and cDNA used for quantitative PCR by SYBR Green Dye gene expression assay on a Bio-Rad CFX96 Real time system. mRNA levels are shown as fold-increase over uninfected mice; n = 10/group. For fecal bacteria detection, PowerSoil DNA Isolation Kit (MO BIO Laboratories, Carlsbad, CA) was used to extract total DNA according to the manufacturer’s instructions. Real-time PCR analyses were performed on 2 ng of total DNA template (SsoAdvanced SYBR Green Supermix, Bio-Rad) to target bacteria group-specific 16S rDNA sequences, as previously described [17]. Groups tested include Enterobacteriaceae family (Proteobacteria phyla) and *Bifidobacterium* genus (Actinobacteria phyla). Specific groups were normalized to the housekeeper Eubacteria group; n = 10/group. For bacterial dissemination, total DNA from murine organs was extracted by using gDNA MiniPrep kit (Zymoresearch, Irvine, CA). Ten ng of total DNA was used to assess the dissemination of *Bifidobacterium* and Enterobacteriaceae; n = 6/group. A list of primers used and their sequences can be found in Table S1. For statistical analyses of gene array data, unpaired *t-*tests were performed using the relative expression levels of each gene at the specified time point compared to the relative expression level of the same gene in uninfected mice.

### 16S Ribosomal DNA Sequencing

For microbiome analyses, fecal DNA samples were amplified by Illumina Miseq compatible primers, targeting the 16s rDNA V4–V5 region. Amplicons were purified by QIAquick Gel extraction kit (Qiagene, Madison, WI) and quantified by Qubit 2.0 Fluorometer (Invitrogen, Grand Island, NY) and Kapa SYBR fast qPCR kit (Kapa Biosystems, Inc., Woburn, MA). Equal amounts of amplicons were pooled with 10% of Phix control. Miseq v2 reagent kit (Illumina, Inc., San Diego, CA) was used to run the pooled samples on the Illumina Miseq machine. The Q score of this run was 86.59% and cluster density was 975±61. Data were analyzed, as previously described [18]. Primers used are found in Table S2.

### Colonoscopy of Uninfected and Sterne-infected A/J Mice

Colons of uninfected or day 3 Sterne-infected A/J mice were imaged with a Multi-Purpose Rigid Telescope attached to a TELE PACK X (Karl Storz–Endoscope, Germany). A/J mice were fasted for 6h prior to visualization of the colons of live animals under appropriate anesthetic conditions.

### Propria (LP) Leukocyte Preparation

Colons surgically excised from Sterne-infected and uninfected control A/J mice were flushed with ice-cold PBS to remove fecal contents, cut open longitudinally, washed, and cut into 1 cm pieces. Tissue pieces were incubated with agitation at 37°C for 25 min in 30 mL of RPMI 1640 Glutamine (GIBCO, Life Technologies, Grand Island, New York) containing 5 mM EDTA (Ambion, Life Technologies), 1 mM DTT (Sigma-Aldrich, St. Louis, MO), 10 mM HEPES (GIBCO, Life Technologies), and supplemented with 5% heat inactivated FBS (HyClone, ThermoFisher Scientific, Waltham, MA) to remove intraepithelial lymphocytes and epithelial cells. After incubation, colonic tissues were washed with ice-cold PBS, minced, and incubated at 37°C with pre-warmed DMEM (GIBCO, Life Technologies) containing 0.25 mg/mL collagenase type VII (Sigma-Aldrich), 0.125 U/mL Liberase TM Research Grade (Roche Applied Science, Indianapolis, IN), 10 mM HEPES, 0.1 M CaCl_2_ (Sigma-Aldrich), and 5% FBS (3×10 min digestions). After each digestion, cell suspensions were passed through a strainer, spun down, and resuspended in DMEM supplemented with 5% FBS. Cells obtained from the three digestions were combined and immediately counted for staining and flow cytometry-based analyses.

### Flow Cytometry and Antibodies

To exclude dead cells, single cell suspensions obtained from processed spleens and LPLs were stained with LIVE/DEAD Aqua Dead Cell Stain Kit (Molecular Probes, Life Technologies). Cells were subsequently washed and incubated with Mouse Fc Blocking Reagent (Miltenyi Biotec, Auburn, CA) prior to staining with combinations of the following antibodies or their corresponding isotype controls purchased from eBioscience (San Diego, CA), Biolegend (San Diego, CA), BD Pharmingen, R&D Systems (Minneapolis, MN), or Cell Signaling Technology, Inc. (Danvers, MA): CD45 (30-F11), CD11c (N418), CD11b (M1/70), CD11b (M1/70), F4/80 (BM8), GR1 (RB6-8C5), I-A/I-E MHCII (2G9), CD3 (145-2C11), CD4 (RM4–5), CD8 (53–607), PD-1/Rat IgG2a, κ, Pro-IL-1β (NJTEN3)/Rat IgG1, κ, TNFα (MP6-XT22)/Rat IgG1, κ, IL-6 (MP5-20F3)/Rat IgG1, κ, IFNγ (XMG1.2)/Rat IgG1, κ, FoxP3 (FJK-16A)/Rat IgG2a, κ, phospho-p38 MAPK (28B10)/Mouse IgG1, phospho-p44/42 MAPK (Erk1/2) (D13.14.4E)/Rabbit. Prior to intracellular staining, cells were fixed and permeabilized with BD Cytofix/Cytoperm (BD Biosciences). Colonic T cells were stimulated with phorbol 12-myristate 13-acetate [(PMA) 50 ng/mL] and ionomycin (1 µg/mL) for 2.5 hrs for the detection of intracellular cytokines. After staining, a BD LSRFortessa (BD Biosciences) cell analyzer was used to acquire fixed cells. Data were analyzed with FlowJo software (Tree Star, Ashland, OR).

### *Ex vivo* Evaluation of MAPKs

Colonic cells were isolated as described above. Equilibrated LP cells were incubated with 1 multiplicity of infection (MOI) of spores or left untreated for 1, 3 and 6 h. Cells were stained and analyzed, as described above.

### Sera Analyses

Cytokines in the sera of Sterne-infected and uninfected A/J mice were measured using the Bio-Plex Pro Mouse Cytokine 23-plex immunoassay kit (Bio-Rad, Hercules, CA). Magnetic beads were acquired with a MAGPIX system (Luminex, Austin, TX) and data analyzed with Bio-Plex Data Pro Software (Bio-Rad).

### Statistical Analyses

Unless stated otherwise, representative data indicate mean ± SEM. Significance was determined by two-tailed unpaired *t-*tests for two group comparisons (GraphPad Prism 5 for Mac OS X, La Jolla, CA).

## Results

### Lethality and Systemic Dissemination of GI *B. anthracis* Infection

**Figure 1:**
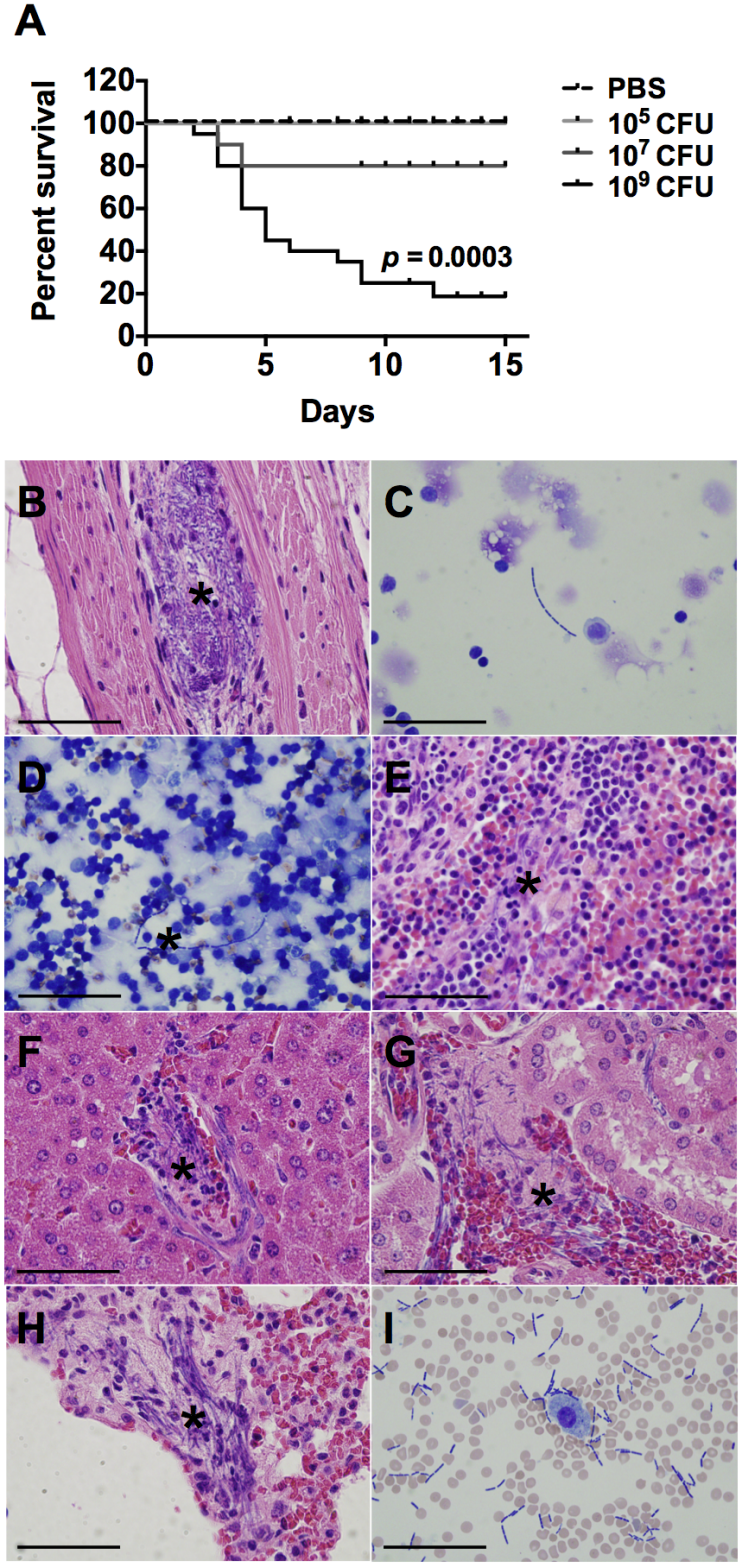
Lethality and Systemic *B. anthracis* Sterne Dissemination in A/J Mice. A. A/J mice were orally gavaged with 10^5^, 10^7^, or 10^9^ spores of the Sterne strain of *B. anthracis*. Lethal infection was established within 3 days in A/J mice receiving 10^9^ spores; n = 10 mice/group (10^5^ and 10^7^), n = 20 mice/group (10^9^). Experiments were performed a minimum of three times. Statistical significance was calculated using the log-rank test. After 3 days of infection, A/J mice were sacrificed; both spores and vegetative bacilli (marked with *) were observed in the colon (**B**), MLNs (**C**), spleen (**D**, **E**), liver (**F**), kidneys (**G**), lungs (**H**), and in bronchoalveolar lavage (BAL) fluid (**I**). Bar = .

To explore GI anthrax pathogenesis while circumventing the difficulties of working with biosafety level 3 (BSL3) *B. anthracis* strains, we employed an extensively used mouse model, A/J mice, which is highly susceptible to *B. anthracis* Sterne [19]. Given the route of infection utilized in our studies, and the potential excretion of gavaged Sterne spores, doses that have been previously shown to be lethal in experimental models of inhalational anthrax [20] resulted in little to no death of A/J mice when administered orally. The lethal dose 50 (LD_50_) for this bacterium via the respiratory route of infection has been reported to be as low as 1×10^3^ CFU of spores/mouse [21], and the LD_50_ of the vegetative bacteria was previously found to be 2.3×10^7^ CFU [13]. However, to better mimic the likely form of *B. anthracis* when ingested, we fed A/J mice varying doses of Sterne spores. We found that 10^5^ spores given orally did not kill any mice and inoculation with 10^7^ spores resulted in 80% survival, likely because of the myriad of enzymes and the extreme pH levels of the gut milieu. The oral LD_50_ was approximately 5×10^8^ spores/mouse (Fig. 1A), and 80% of infected mice died when orally gavaged with 10^9^ Sterne spores (Fig. 1A). These mice were lethargic and showed signs of dyspnea as early as 2 days post-infection. After 3 days of infection, long chains of vegetative bacilli with the characteristic “bamboo-like appearance” were observed (marked with asterisk for ease of visualization) not only in the colon (Fig. 1B), but also systemically (Fig. 1C–I). Vegetative bacilli were seen in numerous organs, including the mesenteric lymph nodes (MLNs) (Fig. 1C), spleen (Fig. 1D, E), liver (Fig. 1F), kidneys (Fig. 1G), lungs (Fig. 1H), and in BAL fluid collected from the lungs (Fig. 1I). In some mice, vegetative bacilli were found in the liver after only 1 day of infection (Fig. S1A), suggesting a hematogenous component in the dissemination process, considering the portal vein carries blood from the GI tract to the liver for nutrition and detoxification. Anthrax-induced death in A/J mice is thought to be due to bacteremia and toxemia resulting from the high number of *B. anthracis* microbes in the periphery [19]. Indeed, we noted focal and diffuse areas of lympholysis in the spleens of infected mice (Fig. 1D, E), consistent with the known necrotizing effects of the toxins. Establishment of active infection is required for morbidity and mortality in our model, as A/J mice that were given LT (125 µg) orally did not show any signs of illness, likely due to proteolytic enzymes within the GI tract (Fig. S1B). In contrast, the same LT dose, when given i.p., resulted in the death of over 50% of the mice (Fig. S1B). Nonetheless, both routes of injection resulted in decreased expression of interleukin (IL)-1β in the colonic tissues (Fig. S1C).

### GI Epithelial Barrier Dysfunction and Dysbiosis in Sterne-infected A/J Mice

**Figure 2:**
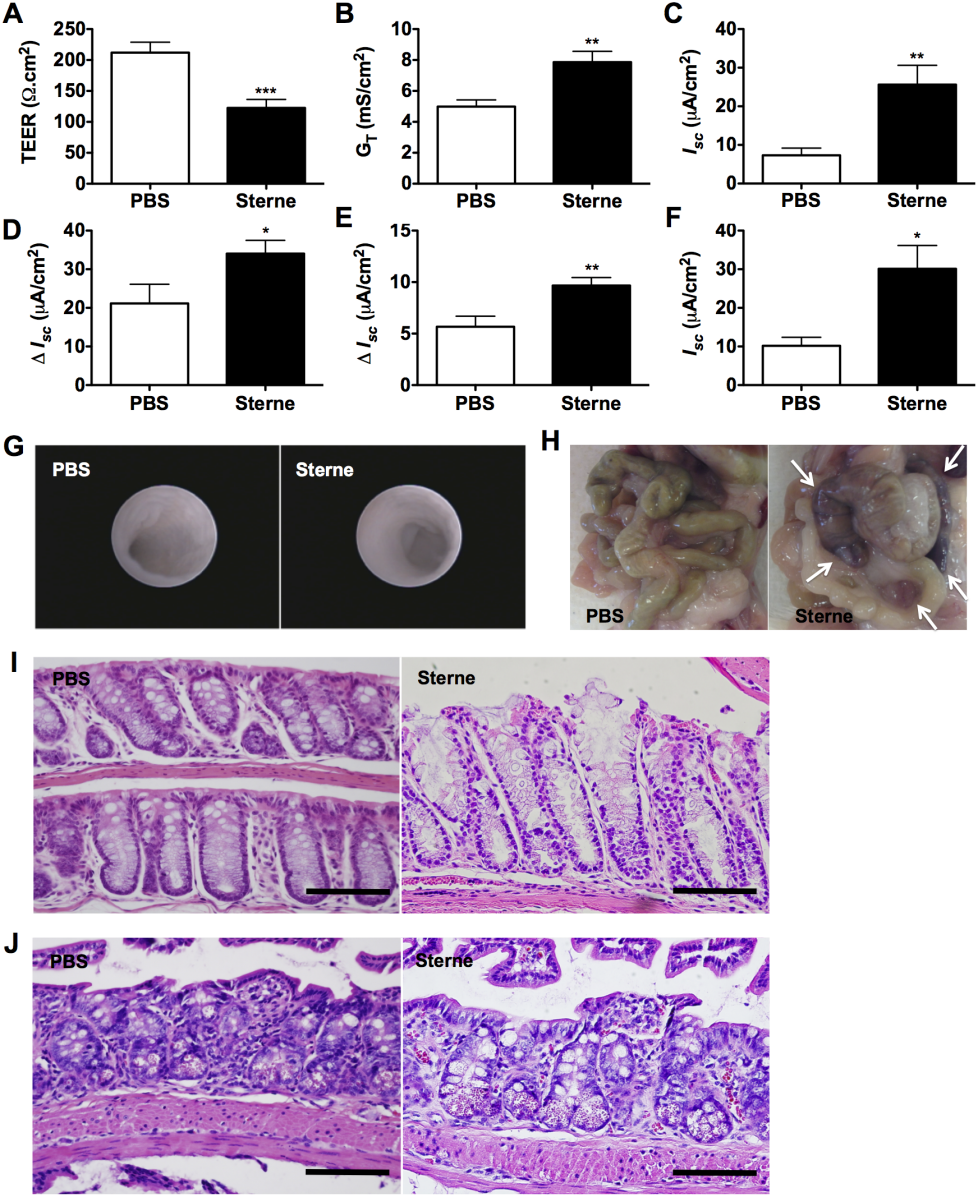
GI Epithelial Barrier Dysfunction Induced by Sterne Infection. A/J mice were orally gavaged with 10^9^ spores of the Sterne strain of *B. anthracis* and intestinal barrier integrity was analyzed *ex vivo* three days post-infection; n = 5 mice/group. TEER (**A**), trans-epithelial conductance (**B**), and short-circuit current (**C**) of Sterne-infected versus uninfected A/J mice. **D and E**. Delta current (Δ*I_SC_*) before and after cholinergic (**D**) and histamine (**E**) challenges. **F**. Post-challenge secretory current of Sterne-infected versus uninfected A/J mice. Data are shown as mean +/− SEM. *P<0.05, **P<0.01, ***P<0.001 compared with PBS. **G**. Colonoscopies were performed in groups of uninfected and 10^9^ Sterne spores-infected A/J mice three days post infection with a Multi-Purpose Rigid Telescope attached to a TELE PACK X. **H**. Gross hemorrhage in the small intestines**.** **I and J.** Hemorrhagic lesions in the colon (**I**) and small intestine (**J**) of Sterne-infected A/J mice. Bar = 200 µm.

Observing the dissemination of *B. anthracis* Sterne in various mouse organs led us to investigate the intestinal barrier function of infected mice. We tested the epithelial barrier integrity of colons isolated from mice infected for 3 days. Infected colons showed a significant breakdown of intestinal barrier integrity, as evidenced by a significantly lower transepithelial electrical resistance (TEER) (Fig. 2A). Consistent with barrier disruption, infected mice also exhibited higher transepithelial conductance (*G_T_*), and short-circuit current (*I_SC_*) (Fig. 2B, C). Additionally, infection altered transcellular ion transport, which is manifested as abnormal electrogenic ion transport responses to cholinergic/muscarinic and histamine challenges (Fig. 2D, E, respectively) and abnormally high post-challenge secretory currents (Fig. 2F). Despite these signs of colonic mucosal damage, colonoscopies performed 3 days post-infection indicated no signs of hemorrhagic lesions within the colons of the majority of the mice studied (Fig. 2G). However, a very small number of mice (<10%) did show gross intestinal hemorrhage (Fig. 2H), vascular congestion, and trace microscopic evidence of hemorrhage in the colons (Fig. 2I) and in the small intestines of infected mice (Fig. 2J).

**Figure 3:**
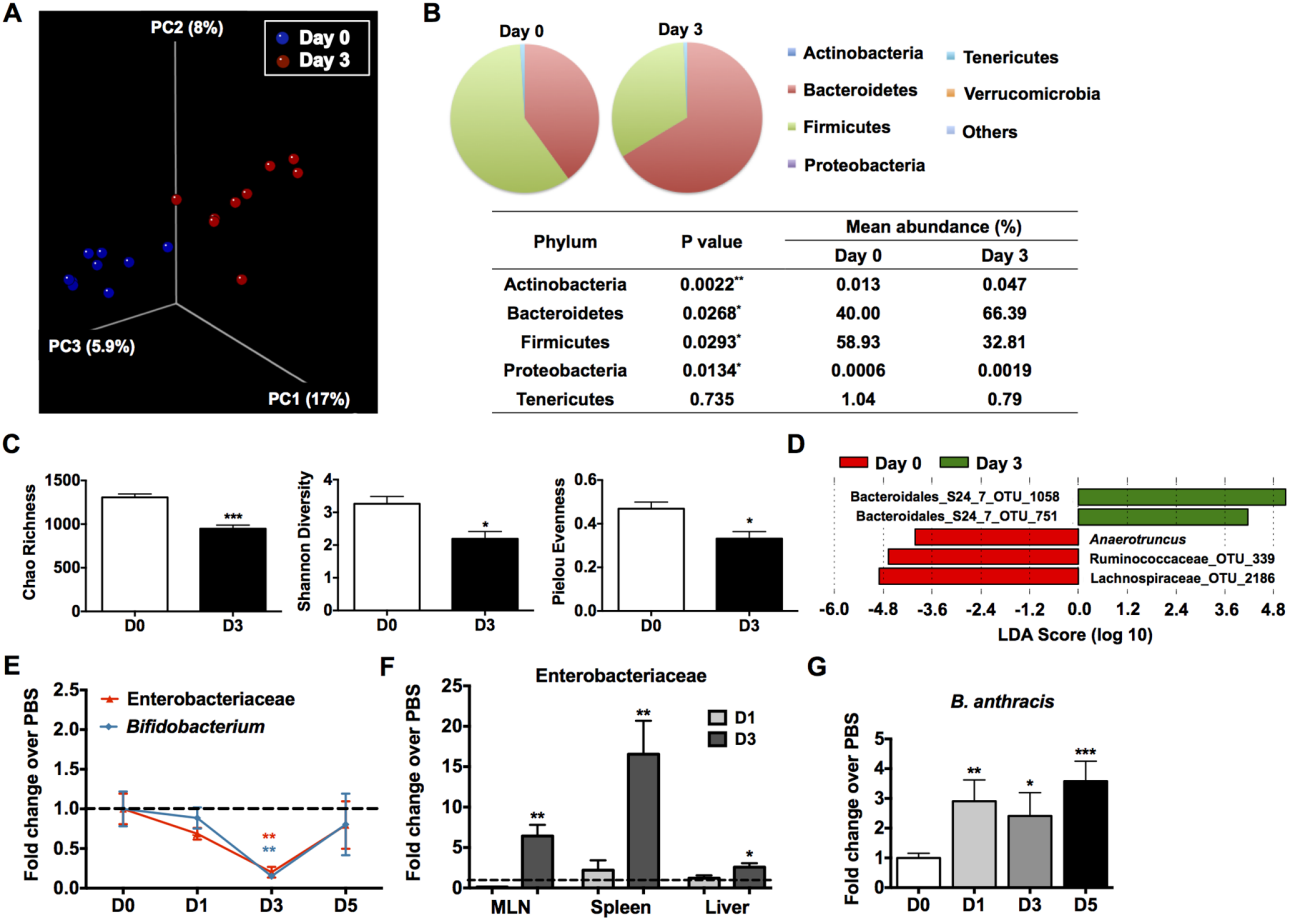
GI Dysbiosis Subsequent to Sterne Infection. A/J mice were orally gavaged with 10^9^ spores of the Sterne strain of *B. anthracis* and changes in microbiota composition during the course of Sterne infection were monitored. **A**. Unweighted UniFrac analyses were used to calculate distances between samples obtained from Sterne-infected A/J mice before infection and three days post-infection and three dimensional scatterplots were generated by using principal coordinate analysis (PCoA); n = 9 mice/group. **B**. Average abundance values of indicated phyla. **C**. Decreased microbial diversity, evenness, and species richness. Left: The Chao richness index was used as a measure of species richness. Middle: The Shannon diversity index was used to estimate microbial diversity for each group. Right: The species evenness index was calculated using the formula J’ = H’/H’_max_, where H’ is the Shannon diversity index and H’_max_ is the maximal value of H’. Data are shown as mean +/− SEM. *P<0.05, ***P<0.001 compared with PBS-treated or day 0 mice. **D**. Bacteria genera most enriched or depleted in Sterne-infected mice at day 3 versus day 0, as measured by linear discriminant analysis (LDA). **E**. Reduced relative abundance of Enterobacteriaceae and *Bifidobacterium* in Sterne-infected A/J mice. **F**. Presence of Enterobacteriaceae in MLNs, spleens, and livers of Sterne-infected mice. **G**. Persistence of *B. anthracis* spores in the feces of Sterne-infected mice.

A mutualistic relationship exists between the intestinal microbiota and the epithelial cells that comprise the single cell barrier between the host and the intestinal lumen, and both populations can modulate the other [22]. Thus, we examined the composition of the gut microbiota of infected mice. In fact, significant changes in the composition of the microbiota were noted in the Sterne-infected mice (Fig. 3). Global changes in microbial community distribution were analyzed by the UniFrac method [23]. In principle coordinate analyses (PCoA), the gut microbes before and 3 days post-infection clustered separately (Fig. 3A), indicating that GI *B. anthracis* Sterne significantly promoted microbial dysbiosis, in which a significant reduction in species richness and changes in the dominant phyla were observed (Fig. 3B, C). Several algorithms were used to determine the species richness and diversity within our samples. For instance, Chao Richness represents an estimate of the total number of species in the samples analyzed as it considers the sequences obtained a subsample of the entire community. Pielou evenness describes how close in numbers species in an environment are; a value nearing 1 indicates an even distribution within the community. Finally, Shannon Diversity takes into account both abundance and evenness within the community.

Subsequently, the algorithm linear discriminant analysis (LDA) effect size (LEfSe) [24] was used to determine which bacteria taxa were differentially represented with infection. Differentially depleted and enhanced genera were mostly composed of unculturable bacteria (Fig. 3D); however, infected mice were enriched for the novel genus *Anaerotruncus*, of which *A. colihominis* is the only described species and has been previously shown to cause bacteremia [25]. Additionally, to overcome PCR primer bias leading to underrepresentation of *Actinobacteria*, in particular bifidobacteria [26], real-time PCR was used to quantify *Bifidobacterium* in the colons. We noted a significant decrease in the relative abundance of *Bifidobacterium* with infection (Fig. 3E); the relative abundance of Enterobacteriaceae was also decreased (Fig. 3E). Because of the breach in intestinal epithelial integrity with infection, we then determined the presence of Enterobacteriaceae systemically. Indeed, members of this bacterial family were detected in the spleen, liver, and MLNs of infected mice (Fig. 3F). However, *Bifidobacterium* was not detected in these tissue samples (data not shown), indicating that its reduction in the colon may represent an actual depletion subsequent to infection. Not surprisingly, fecal shedding of Sterne was observed in all infected mice after one day of infection (Fig. 3G).

### Innate Immune Responses in *B. anthracis* Sterne-infected Mice

It was previously demonstrated that innate cells, including dendritic cells (DCs) are the first targets for *B. anthracis* toxins such as LT [27], [28]. The internalized toxin complexes in the cytosol function as Zn^+2^-dependent metalloproteases that cleave members of the mitogen-activated protein kinase kinase (MAPKK) family, consequently blocking critical signals for cell activation [29]–[33], all of which could contribute to a failure in microbial clearance [28], [34], [35]. In line with these findings, it was recently observed that both toxins, LT and ET, significantly impair protective innate immune responses [28], [34]–[36].

**Figure 4:**
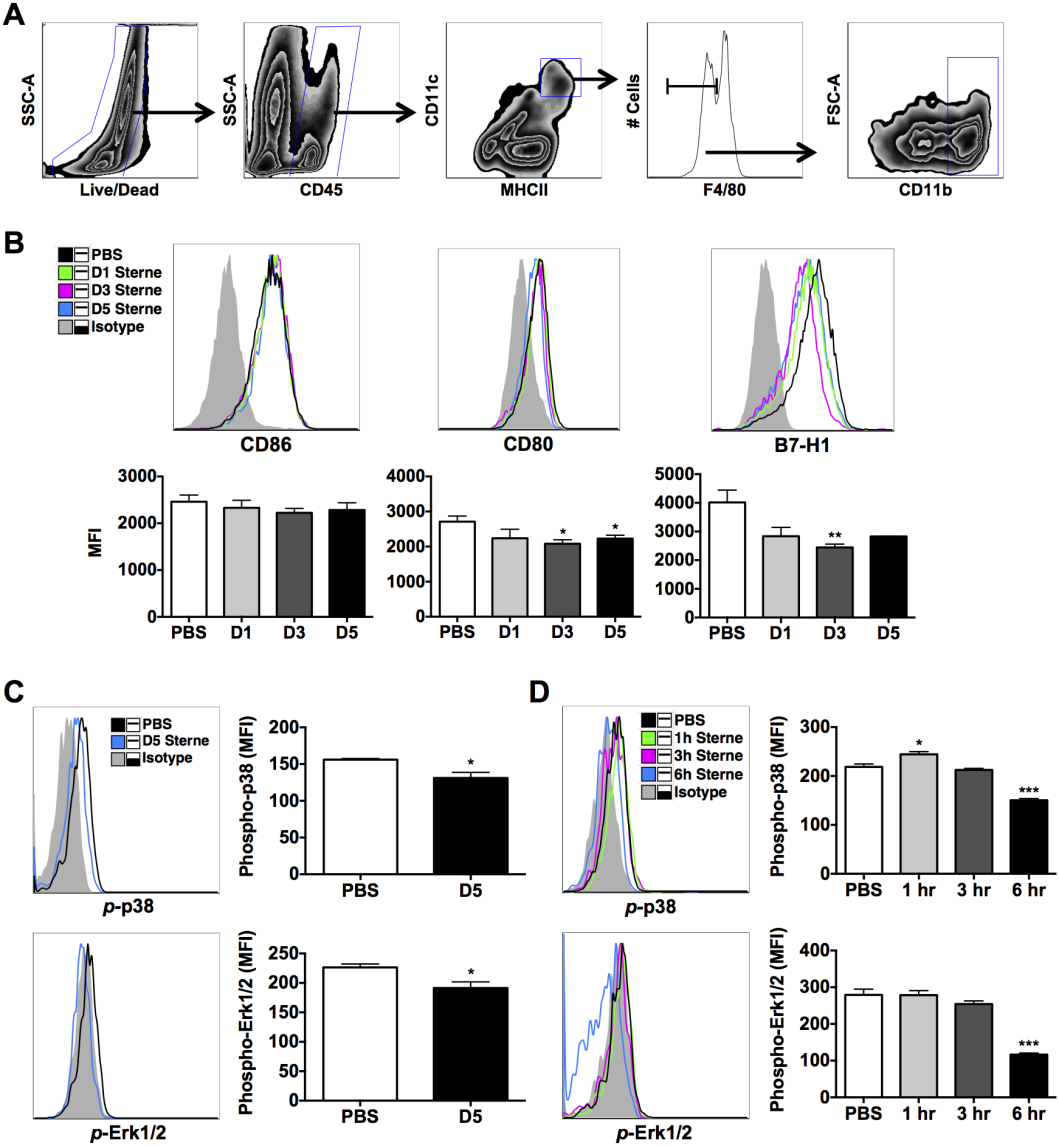
Cleavage of MAPKs in DCs of Sterne-Infected A/J Mice. A/J mice were orally gavaged with 10^9^ spores of the Sterne strain of *B. anthracis* and DCs analyzed at various time points. **A**. Gating strategy for the analysis of CD45^+^MHCII^hi^CD11c^+^F4/80^−^CD11b^+^ colonic DCs. **B**. Cell surface expression of CD86, CD80, and B7-H1 by colonic DCs was analyzed by flow cytometry. Gray tinted line = isotype control; black line = PBS group; green line = day 1 Sterne-infected A/J mice; magenta line = day 3 Sterne-infected A/J mice; blue line = day 5 Sterne-infected mice. **C**. Activity of MAPKs in colonic DCs of infected versus uninfected mice was analyzed by flow cytometry. Gray tinted line = isotype control; black line = PBS group; blue line = day 5 Sterne-infected mice. **D**. Colonic LP cells were isolated from uninfected A/J mice and incubated with 1 MOI of *B. anthracis* spores for 1, 3, or 6 hours. Activity of p38 and Erk1/2 was subsequently analyzed in colonic DCs. Gray tinted line = isotype control; black line = PBS group; green line = 1 hour treatment; magenta line = 3 hour treatment; blue line = 6 hour treatment. Data represent observations from three independent experiments and are shown as mean +/− SEM. *P<0.05, **P<0.01, ***P<0.001 compared with PBS.

**Figure 5:**
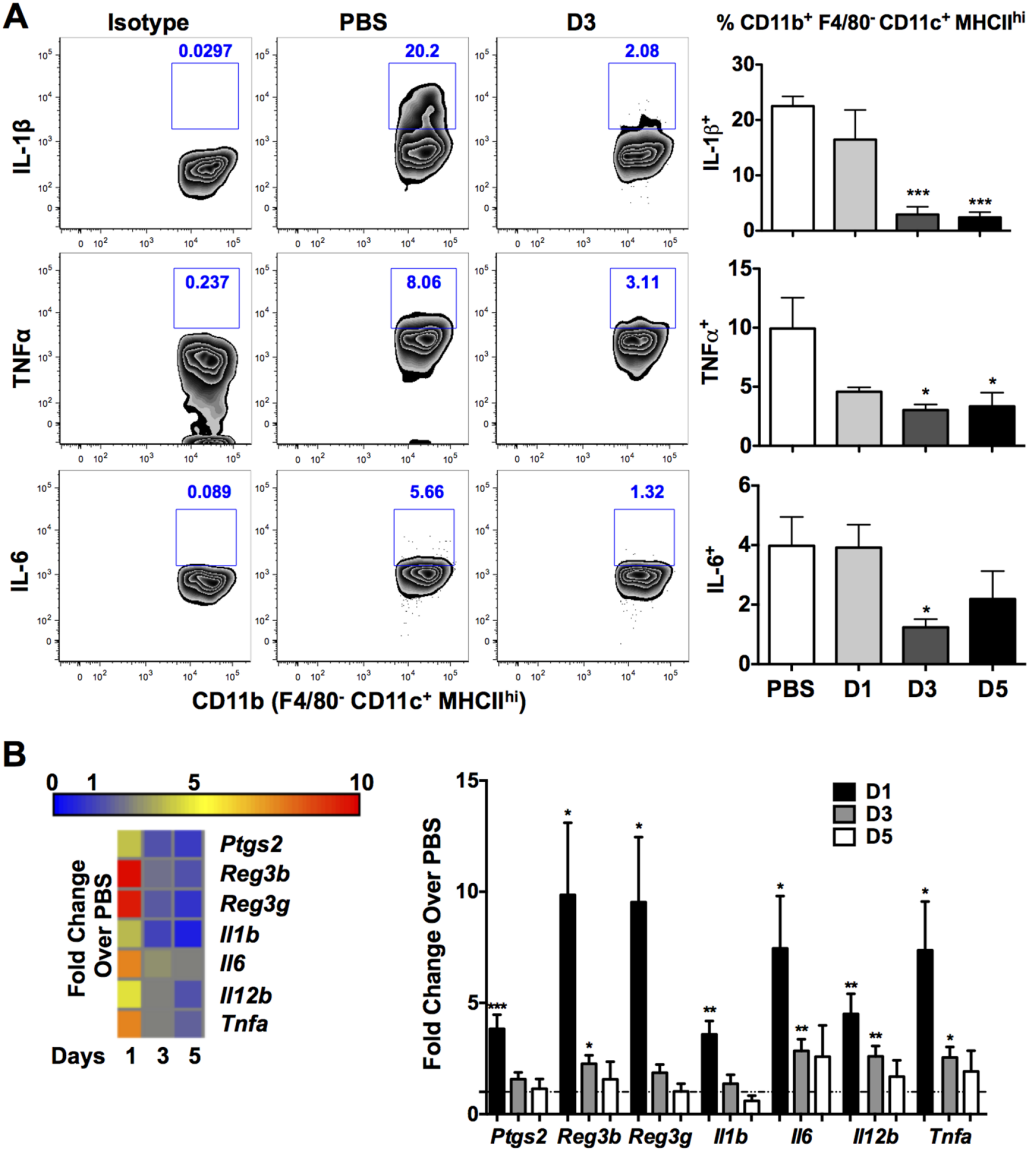
Suppression of Innate Immune Responses in Sterne-Infected A/J Mice. A/J mice were orally gavaged with 10^9^ spores of the Sterne strain of *B. anthracis* and innate immune responses analyzed at various time points. **A**. DCs isolated from the colons of Sterne-infected A/J mice were analyzed by flow cytometry for the production of the pro-inflammatory cytokines IL-1β, TNF-α, and IL-6. Representative plots indicate cytokine production of uninfected and day 3 infected mice. Corresponding isotype controls were utilized for gating of intracellular cytokines. **B**. Gene expression profile of the distal colon of Sterne-infected A/J mice. Data represent observations from four independent experiments and are shown as mean +/− SEM. *P<0.05, **P<0.01, ***P<0.001 compared with PBS.

To demonstrate whether GI anthrax infection induces the same effects in DCs derived from orally infected mice, colonic DCs were isolated and studied. Indeed, the activation of colonic CD45^+^MHCII^hi^CD11c^+^F4/80^−^CD11b^+^ DCs (Fig. 4A), co-stimulatory molecules, CD80/CD86, and co-inhibitory, B7-H1, were unchanged or significantly depressed during infection (Fig. 4B), suggesting inhibition of immune functions. It is worth noting that B7-H1 is tightly regulated by ERK/p38 MAPK signaling [37], which is known to be disrupted by LF [38]. Accordingly, we addressed the question whether GI Sterne infection induces the cleavage of MAPKs in colonic DCs by evaluating the activation of downstream kinases, p38 and Erk1/2, *in vivo* and *ex vivo*. Data clearly show that infection with Sterne resulted in the impairment of p38 and Erk1/2 phosphorylation in colonic DCs of mice that were orally gavaged with 1×10^9^ spores (Fig. 4C). To strengthen this observation, we then isolated and infected colonic cells with 1 MOI of *B. anthracis* Sterne spores for 1, 3, or 6 hours. A transient increase in p38 and Erk1/2 phosphorylation in total colonic cells (Fig. S2) and more specifically phospho-p38 in DCs was observed at earlier time points; however, after 6 hours, cleavage of p38 and Erk1/2 phosphorylation was measured (Fig. 4D, and Fig. S2). Furthermore, *B. anthracis* Sterne also downregulated IL-1β, tumor necrosis factor (TNF)-α, and IL-6 by DCs (Fig. 5A), a consequence that is attributed to cleavage of MAPKs [28], [34], [35].

Having noted inhibition of colonic DC function, transcriptional changes in genes encoding pattern recognition receptors and inflammatory mediators were analyzed in the colonic tissues of infected mice as a measure of local induced inflammation and immune activation by *B. anthracis* Sterne. Inflammation-associated genes were found to be transiently upregulated early during Sterne infection (day 1, Fig. 5B); however, this gene upregulation was rapidly downregulated on days 3 and 5 post-infection, possibly due to the release of bacterial toxins (Fig. 5B). Therefore, the GI milieu mirrored the phenomenon of immune dysfunction observed in colonic DCs. Similar to the lack of colonic innate immune activation observed, systemic innate responses were also suppressed in infected mice, as both splenic DCs and macrophages produced less pro-inflammatory cytokines than those of uninfected mice (Fig. S3A, B). Not unexpectedly, given the aforementioned immune defects, with the exception of IL-1β at day 1 post-infection, no major increases in pro-inflammatory cytokine levels were detected in the sera of infected mice compared to control animals (Fig. S4); instead, we found that circulating levels of interferon (IFN)γ were reduced after 3 days of infection (Fig. S4).

### Local and Systemic T Cell Immune Responses in Sterne-infected A/J Mice

**Figure 6:**
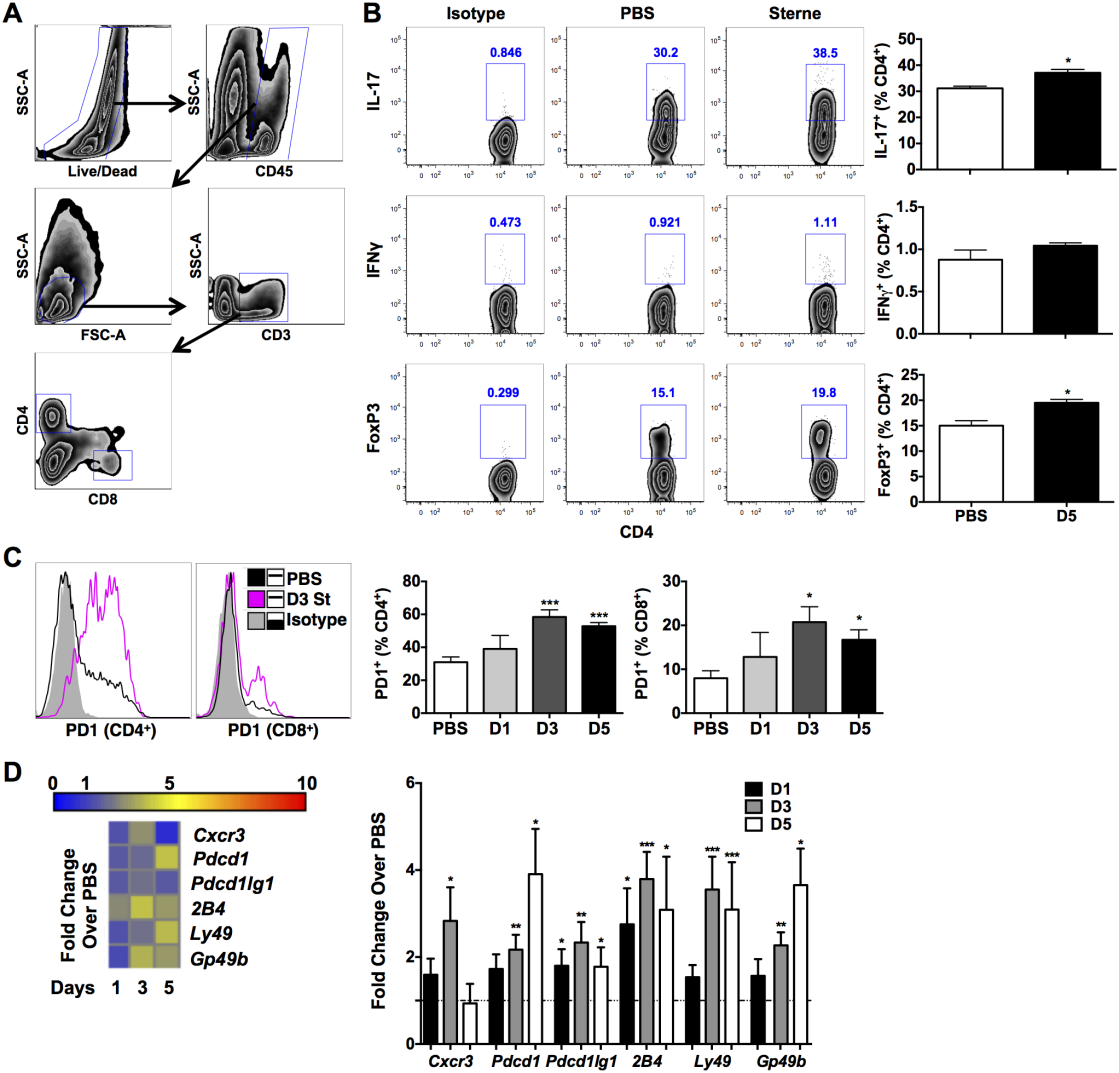
T Cell Responses in Sterne-Infected A/J Mice. A/J mice were orally gavaged with 10^9^ spores of the Sterne strain of *B. anthracis* and adaptive immune responses in the colon analyzed at various time points by flow cytometry. **A**. Gating strategy for the analysis of colonic T cells. **B**. Th1, Th17, and regulatory T cells responses were tested by flow cytometry. Representative plots indicate cytokine production of uninfected and day 5-infected mice. **C**. Surface expression of PD1 in colonic T cells. **D**. Gene expression profile of the distal colon of Sterne-infected A/J mice. Data represent observations from four independent experiments and are shown as mean +/− SEM. *P<0.05, **P<0.01, ***P<0.001 compared with PBS.

To better understand the impact of deteriorated innate cell function resulting from GI anthrax on local and systemic T cell responses, we evaluated Th1, Th17, and regulatory (Treg) T cell activation in the colonic LP (Fig. 6A) and in the spleens of infected mice. Colonic and splenic T cell responses in infected mice were mostly limited to the CD4^+^ T cell subset. No significant changes were observed in day 1 and day 3-infected mice (Data Not Shown). However, a significant increase in the frequency of IL-17A^+^CD4^+^ T cells (day 5) was noted in the colons of Sterne infected mice (Fig. 6B, top). Systemically, splenic CD4^+^ T cells from infected mice showed increased intracellular IFNγ and a transient increase in IL-17A^+^CD4^+^ T cells (Fig. S5A, B). Recently, it has been shown that depending on the levels of ET, *B. anthracis* can suppress T cell proliferation, skew CD4^+^ T cells toward Th17 differentiation, and/or potentiate Th2 polarization [39]. Moreover, concurrent with increased colonic Th17 responses, Tregs were also enhanced in day 5-infected mice (Fig. 6B, bottom), suggesting that these induced levels of Tregs might potentially regulate protective Th17 immunity, which has been demonstrated to be critical for protection in inhalational anthrax infection [40].

Increased levels of Programmed Death-1 (PD-1) on T cells has recently been shown to be a critical “molecular signature” of T cell exhaustion in several models of chronic viral infection; this “molecular signature” also includes increased mRNA for cell-surface receptors suspected or known to have inhibitory function, such as 2B4, Ly49 family members, and GP49B [41]. Consistent with induction of immune dysfunction by GI anthrax infection, PD-1 receptor expression was significantly augmented on colonic CD4^+^ and CD8^+^ T cells of infected mice (Figure 6C), the transcription of which was also confirmed by gene expression analyses (Fig. 6D). We also observed increased gene expression of *Gp49b*, *Ly49*, and the *2B4* short isoform (Fig. 6D) in colonic tissues derived from *B. anthracis* infected mice. Moreover, colonic expression of *Cxcr3*, a chemokine receptor that is preferentially expressed on Th1 cells and promotes their recruitment to sites of inflammation [42], [43], was significantly increased (∼3 fold) in day 3-infected mice (Fig. 6D); however, expression of this critical chemokine receptor was significantly down-modulated by day 5 of infection, highlighting the change in the inflammatory status of the gut upon GI anthrax progression.

## Discussion

Contact with *B. anthracis*-infected animals or consumption of tainted, undercooked meat may result in GI anthrax in humans [13], [44]. In recent years, advances have been made toward the development of experimental models to study the pathogenesis of GI *B. anthracis* infection and the effects of its toxins on GI health [11]–[13], [45]. Unlike direct intragastric infection with *B. anthracis* spores [11], [12], oral gavage with Sterne spores did not induce severe pathology in the small intestine, and the Peyer’s Patch did not appear to be the primary site of *B. anthracis* growth. Moreover, intragastric infection with vegetative bacilli resulted in rapid morbidity and mortality of the mice [13]. Such morbidity leads to significant nutritional deficiencies in the animals, which have known negative immunologic consequences [46]. Therefore, our model of GI anthrax provides a unique window of moderate disease, whereby the immunologic status of the animals better indicates responses to the pathogen itself. To gain a better understanding of GI anthrax pathogenesis, A/J mice were orally infected with 10^9^ *B. anthracis* Sterne spores. Bacterial dissemination was observed in various visceral organs as early as one day post-infection (liver), which potentially contributed to further systemic bacterial pathogenesis resulting in impaired immunity, whereupon mice succumbed to infection as early as day 2 post-infection. Consequently, our findings provide in-depth immunologic studies as well as detailed investigations of the intestinal health of infected mice as it relates to its barrier function and microbial composition.

Emerging data suggest that *B. anthracis* toxins suppress host immunity, allowing the pathogen to significantly replicate, resulting in septic shock [47]. We posit that, as is the case with many other microbial pathogens, the first event is the ability of *B. anthracis* to disrupt epithelial barrier function via synergy of LT with *B. anthracis* S-layer protein A (BslA) [32], as this protein has been identified as an important surface adhesin of this deadly pathogen [48]. Disruption of intestinal barrier function undoubtedly contributes to bacterial dissemination locally and systemically, a mechanism that may change the composition of the gut microbiota, as significant decreases in the relative abundance of *Enterobacteriaceae* and *Bifidobacterium* in the feces of Sterne infected mice were observed. We also detected *Enterobacteriaceae* members in two important peripheral immune organs, the MLNs and the spleen. The observed bacterial translocation and dissemination could be seen as a potential cause for the lower numbers in the feces of infected mice; however, further studies are warranted to ascertain this possibility. On the other hand, *Bifidobacterium*, typically recognized as a beneficial bacterial group in the host gut, was not detected in any other organ, suggesting that this group was potentially out-competed for survival in the altered intestinal microenvironment upon *B. anthracis* Sterne infection. Indeed, this has been shown to be the case with many patients with colorectal inflammatory diseases, since *Bifidobacteria* protect the mucosae from damaging inflammation [49] and enhance barrier function [50]. When considering the concomitant intestinal barrier damage caused by *B. anthracis* infection, the dissemination of *Enterobacteriaceae* may have contributed to pathogenic inflammation, while elimination of the beneficial bacterium, *Bifidobacterium,* may have left the host with less regulatory mucosal responses involved in healthy gut homeostasis.

PCoA demonstrated a significantly altered gut microbial composition in mice after 3 days of infection, indicating a global change in the gut flora at the phylum level. Uncultured *operational taxonomic units* (OTU) 751 and OTU 1058 were shown to be dramatically increased, along with *Anaetruncus*, while uncultured OTU 339 and OTU 2186 were decreased with infection. Both unculturable OTU 751 and OTU 1058 are classified as part of the Bacteroidales S24-7 family, which has been found to be a dominant uncultured family in the gut whose function is likely the predominant contributor to the Bacteroidetes phylum expansion. Unculturable OTU 339 and OTU 2186 can be classified into the Ruminococcaceae family and Lachnospiraceae family, respectively. Depletion of both of these two families has consistently been associated with colonic inflammation [51]. Most of these two families’ members belong to *Clostridium* cluster IV and XIVa, which have the potential to induce butyrate production [52], Tregs [53], maintenance of barrier function [52], and competition with other pathogens in the colonization of the gut. Therefore, the collective depletion of these commensal bacteria in the gut may have negatively affected the local immunity by promoting pathogenic inflammation and disrupting mucosal homeostasis.

Controlled inflammatory responses are necessary to promote protective immunity and overcome pathogen challenge after infection. However, certain pathogens have evolved to evade immune recognition and clearance through NF-κB and MAPK signaling inhibition [54]. Accordingly, one day post-infection we found a robust increase in the transcription of genes involved in innate immune recognition (Data Not Shown); however, expression of these genes rapidly decreased to basal levels, and in certain cases, was even lower than those of the controls, suggesting suppression of critical sensing molecules to mobilize protective immunity against pathogens. Most of the genes whose expression were upregulated are MAPK-dependent, indicating that within one day endospores germinate into the vegetative form to secrete LT, which likely causes immune suppression. Indeed, infected mice exhibited global immune suppression, as infection did not elicit protective immune activation in either colonic or splenic DCs. This is consistent with previous reports showing that LT suppresses IL-6 and TNF-α in DCs by disrupting MAPK signaling [28]. Suppression is thought to occur early in infection, while in later stages, the toxin exerts inflammatory effects contributing to toxic shock and bacteremia [28]. Our studies clearly demonstrate the inhibition of MAPK signaling in intestinal immune cells (e.g., DCs) upon GI anthrax infection.

The gut microenvironment is tightly controlled by innate cells that potentially impact T cell function by regulating PD-1/B7-H1 interactions, which may result in T cell “exhaustion” as a consequence of infection [55]. Importantly, it has been shown that the MAPK-dependent transcription factor, T-bet [56], is a negative regulator of PD-1 [57]. Thus, inhibition of MAPK-activity by LT may have downregulated T-bet activation, resulting in the increased expression of PD-1 on T cells. While the expression of B7-H1 was reduced on DCs [58], B7-H1 transcription was globally upregulated in colonic cells other than DCs. Furthermore, MAPK-dependent cytokines (e.g., IL-1β, TNF-α, and IL-6) were downregulated in DCs and macrophages as a result of Sterne infection. In the colon, expression of *Cxcr3,* which is a signature chemokine receptor for IFNγ^+^ Th1 cell recruitment, was transiently increased with a significant reduction by day 5, indicating a potential lack of supporting immune mechanisms to induce microbial protective immunity in the GI tract. Conversely, in the spleen, the immune environment was shifted toward a mixed Th1/Th17 response, with increased IFNγ and IL-17A-producing CD4^+^ T cells, a hallmark of bacterial infection [59].

In summary, GI infection with *B. anthracis* is quite different and more complex than infection via the respiratory route. Respiratory surfaces maintain an anti-inflammatory environment [60], as does the GI tract [61]; however, the microbial load of the GI tract is considerably larger and more complex. Respiratory infection with *B. anthracis* has a higher fatality rate and requires smaller inoculums than GI infection, perhaps due to the presence of mitigating factors in the latter, including proteolytic enzyme activity, the extensive gut microbiota, and peristaltic expulsion of spores from the GI tract. Additionally, the breach in gut barrier function caused by *B. anthracis* and its gene products leads to translocation of *B. anthracis* and other gut microbes, which may engage complex signaling events, locally and systemically. Translocation of the gut microbiota could be beneficial to the host by mounting a Th1/Th17 response to clear the bacteria; conversely, it may cause bacteremia and septic shock. The precise contribution of this shift in the composition of the gut microbiome to the pathobiology of GI anthrax has yet to be examined. An early distinction between mice that survive and those that succumb to infection and a better understanding of early innate immune cell activation that bypasses suppressive attempts by *B. anthracis* will be critical in the prevention of fatal systemic disease in animals and in humans.

## Supporting Information

Figure S1 **Morbidity and Mortality in GI Anthrax is Dependent on Active Infection and Involves Hematogenous Spread of Infection.** **A**. A/J mice were orally gavaged with 10^9^ spores of the Sterne strain of *B. anthracis*. One day post-infection, *B. anthracis* Sterne bacilli could be found within the liver of some mice Bar = 50 µm. **B**. A/J mice were orally gavaged with 125 µg LT (PA+LF) or injected intraperitoneally (i.p.) and monitored for morbidity and death. **C**. IL-1β expression profile of the distal colons of A/J mice that were given LT (125 µg) by the oral route versus i.p. injection.
(TIFF)

Figure S2 ***Ex vivo*** **Inhibition of MAPKs in Immune Cells by Sterne.** Colonic LP cells were isolated from uninfected A/J mice and incubated with 1 MOI of *B. anthracis* spores for 1, 3, or 6 hours. Activity of p38 and Erk1/2 was subsequently analyzed with phosphorylation-specific antibodies by Western blot.
(TIFF)

Figure S3 **Splenic Innate Immune Responses in Sterne-infected A/J Mice.** A/J mice were orally gavaged with 10^9^ spores of the Sterne strain of *B. anthracis* and innate immune responses analyzed at various time points by flow cytometry. Splenic DC (**A**) and macrophage (**B**) functions were inhibited after infection as measured by IL-1β and TNF-α production. n = 10 mice/group. Data represent observations from four independent experiments and are shown as mean +/− SEM. **P<0.01, ***P<0.001 compared with PBS.
(TIFF)

Figure S4 **Sera Cytokine Levels of Sterne-infected A/J Mice.** A/J mice were orally gavaged with 10^9^ spores of the Sterne strain of *B. anthracis* and sera collected. Cytokines in the sera of Sterne-infected and uninfected A/J mice were measured using the Bio-Plex Pro Mouse Cytokine 23-plex immunoassay kit. Data are shown as mean +/− SEM; each symbol represents one mouse. *P<0.05, ***P<0.001 compared with PBS.
(TIFF)

Figure S5 **Splenic T Cell Responses in Sterne-infected A/J Mice.** A/J mice were orally gavaged with 10^9^ spores of the Sterne strain of *B. anthracis* and Th1 (**A**) and Th17 (**B**) responses analyzed at various time points by flow cytometry. n = 10 mice/group. Data represent observations from four independent experiments and are shown as mean +/− SEM. *P<0.05 compared with PBS.
(TIFF)

Table S1 List of primer sequences for Real-Time PCR analyses.
(DOCX)

Table S2 List of primer sequences for 16S rDNA analyses.
(DOCX)
