# Supplementary figures and images for: Colonic Immune Suppression, Barrier Dysfunction, and Dysbiosis by Gastrointestinal Bacillus anthracis Infection
Source: PLoS One. 2014 Jun 19;9(6):e100532. doi: 10.1371/journal.pone.0100532 (PMC4063899; doi:10.1371/journal.pone.0100532)

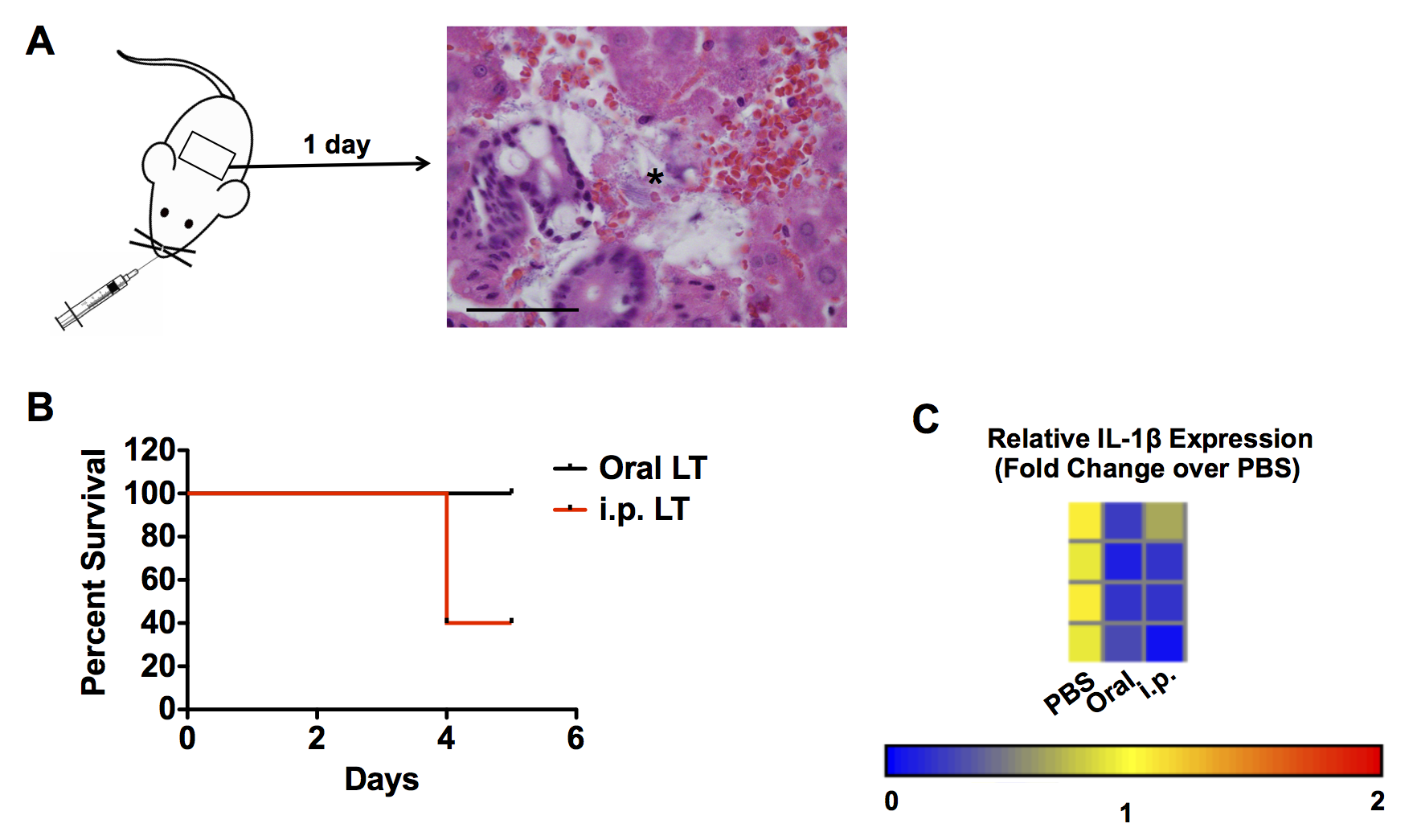

Supplement: Figure S1 — Morbidity and Mortality in GI Anthrax is Dependent on Active Infection and Involves Hematogenous Spread of Infection. A. A/J mice were orally gavaged with 109 spores of the Sterne strain of B. anthracis. One day post-infection, B. anthracis Sterne bacilli could be found within the liver of some mice Bar = 50 µm. B. A/J mice were orally gavaged with 125 µg LT (PA+LF) or injected intraperitoneally (i.p.) and monitored for morbidity and death. C. IL-1β expression profile of the distal colons of A/J mice that were given LT (125 µg) by the oral route versus i.p. injection. (TIFF) [file pone.0100532.s001.tif]

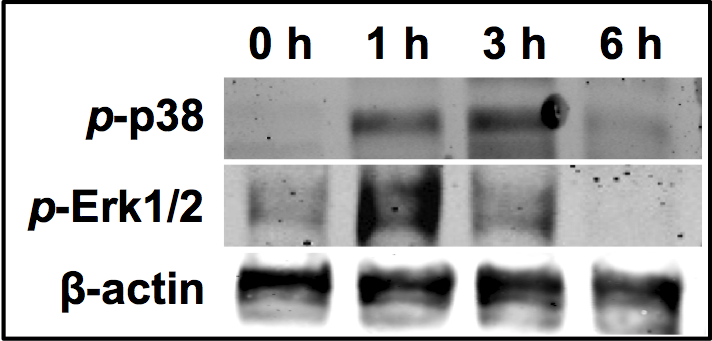

Supplement: Figure S2 — Ex vivo Inhibition of MAPKs in Immune Cells by Sterne. Colonic LP cells were isolated from uninfected A/J mice and incubated with 1 MOI of B. anthracis spores for 1, 3, or 6 hours. Activity of p38 and Erk1/2 was subsequently analyzed with phosphorylation-specific antibodies by Western blot. (TIFF) [file pone.0100532.s002.tif]

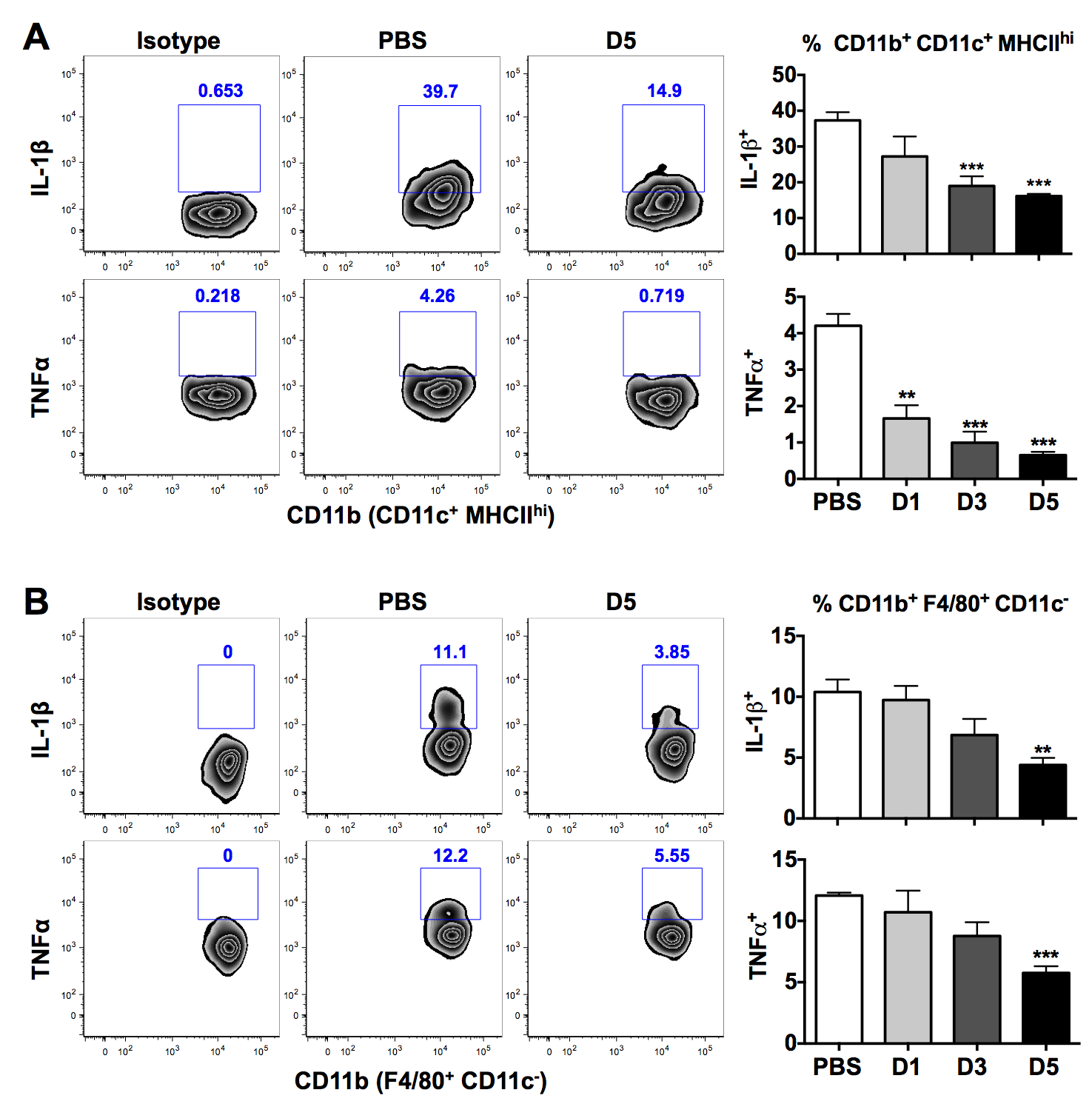

Supplement: Figure S3 — Splenic Innate Immune Responses in Sterne-infected A/J Mice. A/J mice were orally gavaged with 109 spores of the Sterne strain of B. anthracis and innate immune responses analyzed at various time points by flow cytometry. Splenic DC (A) and macrophage (B) functions were inhibited after infection as measured by IL-1β and TNF-α production. n = 10 mice/group. Data represent observations from four independent experiments and are shown as mean +/− SEM. **P<0.01, ***P<0.001 compared with PBS. (TIFF) [file pone.0100532.s003.tif]

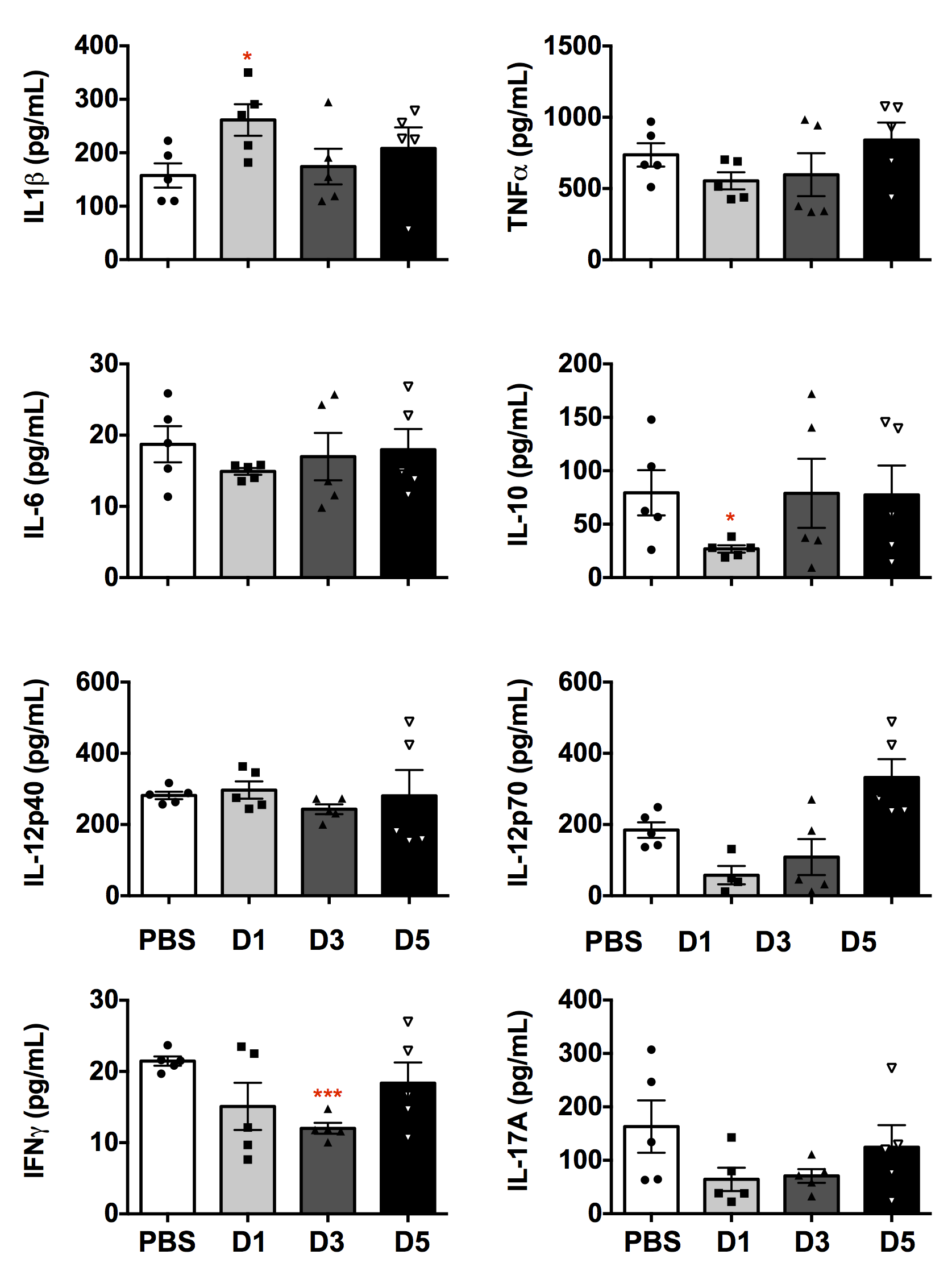

Supplement: Figure S4 — Sera Cytokine Levels of Sterne-infected A/J Mice. A/J mice were orally gavaged with 109 spores of the Sterne strain of B. anthracis and sera collected. Cytokines in the sera of Sterne-infected and uninfected A/J mice were measured using the Bio-Plex Pro Mouse Cytokine 23-plex immunoassay kit. Data are shown as mean +/− SEM; each symbol represents one mouse. *P<0.05, ***P<0.001 compared with PBS. (TIFF) [file pone.0100532.s004.tif]

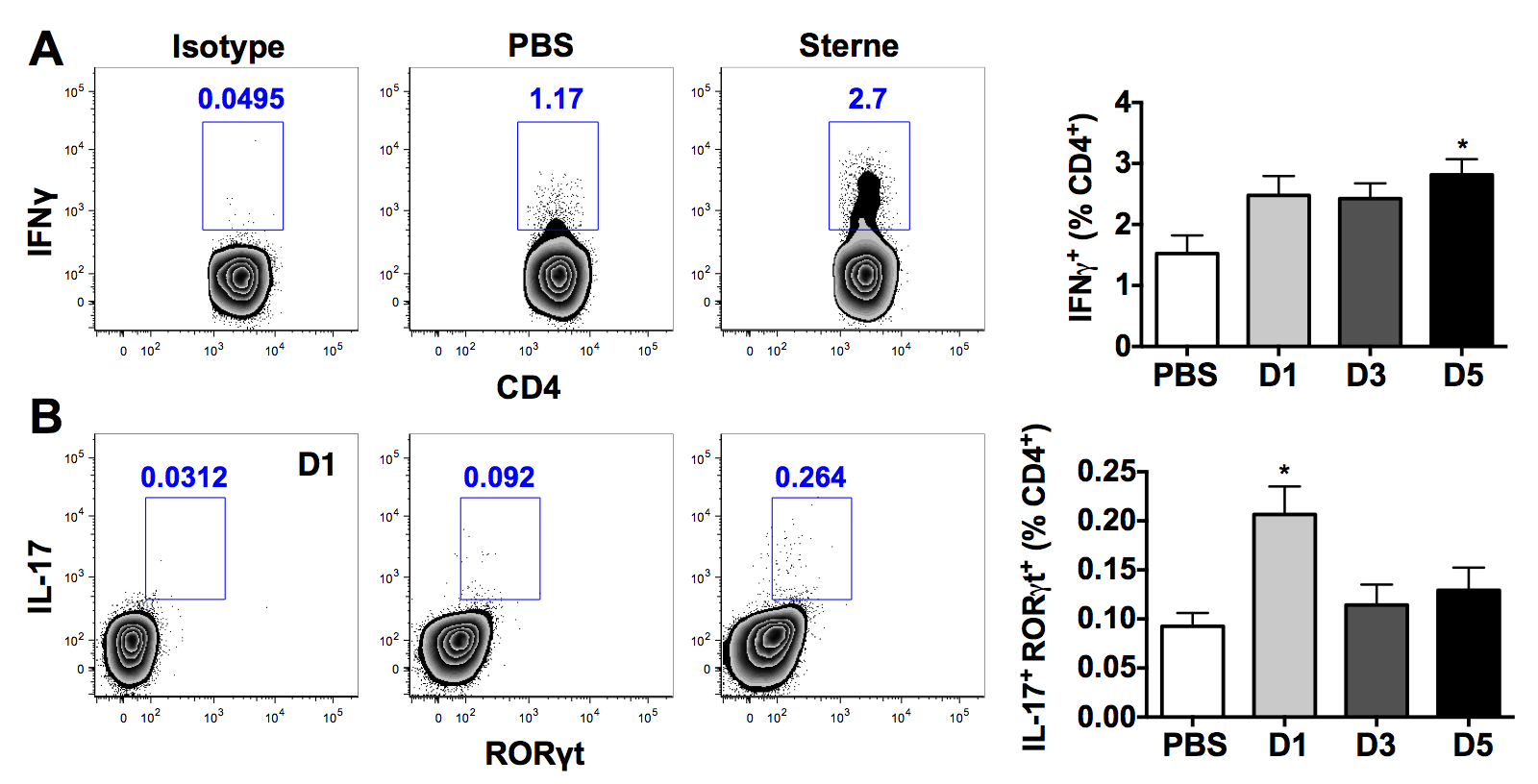

Supplement: Figure S5 — Splenic T Cell Responses in Sterne-infected A/J Mice. A/J mice were orally gavaged with 109 spores of the Sterne strain of B. anthracis and Th1 (A) and Th17 (B) responses analyzed at various time points by flow cytometry. n = 10 mice/group. Data represent observations from four independent experiments and are shown as mean +/− SEM. *P<0.05 compared with PBS. (TIFF) [file pone.0100532.s005.tif]
