# Supplementary material for: Colonic Immune Suppression, Barrier Dysfunction, and Dysbiosis by Gastrointestinal Bacillus anthracis Infection
Source: PLoS One. 2014 Jun 19;9(6):e100532. doi: 10.1371/journal.pone.0100532 (PMC4063899; doi:10.1371/journal.pone.0100532)
Supplement: Table S1 — List of primer sequences for Real-Time PCR analyses. (DOCX) [file pone.0100532.s006.docx]

Table S1

| **Primer** | **Sequence 5’-3’** |
| --- | --- |
| *Ptgs2* For | TGGGTTCACCCGAGGACTGG |
| *Ptgs2* Rev | TCGCACACTCTGTTGTGCTCC |
| *Reg3b* For | CCCTCCGCACGCATTAGTTG |
| *Reg3b* Rev | GCCTCAGCGCTATTGAGCAC |
| *Reg3g* For | CTGCCAAAAGAGGCCCTCAG |
| *Reg3g* Rev | CCCATCCACCTCTGTTGGGTT |
| *Il1b* For | AAGGAGAACCAAGCAACGAC |
| *Il1b* Rev | GAGATTGAGCTGTCTGCTCA |
| *Il6* For | AAA GAG TTG TGC AAT GGC AAT TCT |
| *Il6* Rev | AAG TGC ATC ATC GTT GTT CAT ACA |
| *Il12b* For | CCACTCACATCTGCTGCTCCACAAG |
| *Il12b* Rev | ACTTCTCATAGTCCCTTTGGTCCAG |
| *Tnfa* For | AGTGGTGCCAGCCGATGGGTTGT |
| *Tnfa* Rev | GCTGAGTTGGTCCCCCTTCTCCAG |
| *Cxcr3* For | CTCTTTGCCCTCCCAGATTTC |
| *Cxcr3* Rev | GGCATAGCAGTAGGCCATGAC |
| *Pdcd1* For | ATGTGGGTCCGGCAGGTACCCTGG |
| *Pdcd1* Rev | TCAAAGAGGCCAAGAACAATGTCC |
| *Pdcd1lg1* For | TTCAGATCACAGACGTCAAGCTG |
| *Pdcd1lg1* For | ATTCTCTGGTTGATTTTGCGGTA |
| *2B4* For | ATGTTCAGCTCCCTTCTAG |
| *2B4* Rev | GAGTTCCTCCCTGGCAGATC |
| *Gp49b* For | ACTCACAGCATCAGGCCAAT |
| *Gp49b* Rev | ACTGGTATCCGATGAGGATG |
| *Ly49g* For | GTTGCAGAAACTAGTGAGGAC |
| *Ly49g* Rev | GATGTATGATTTACCACAGTCC |
| *Bifidobacterium* For | CGGGTGAGTAATGCGTGACC |
| *Bifidobacterium* Rev | TGATAGGACGCGACCCCA |
| Enterobacteriaceae For | GTGCCAGCMGCCGCGGTAA |
| Enterobacteriaceae Rev | GCCTCAAGGGCACAACCTCCAAG |
