# Supplementary material for: Colonic Immune Suppression, Barrier Dysfunction, and Dysbiosis by Gastrointestinal Bacillus anthracis Infection
Source: PLoS One. 2014 Jun 19;9(6):e100532. doi: 10.1371/journal.pone.0100532 (PMC4063899; doi:10.1371/journal.pone.0100532)
Supplement: Table S2 — List of primer sequences for 16S rDNA analyses. (DOCX) [file pone.0100532.s007.docx]

**Table S2**

| Samples | Forward primers |
| --- | --- |
| D0 ST-1 | AATGATACGGCGACCACCGAGATCTACACTCTTTCCCTACACGACGCTCTTCCGATCTCGTGTACACCAGCMGCCGCGGTAAT |
| D0 ST-2 | AATGATACGGCGACCACCGAGATCTACACTCTTTCCCTACACGACGCTCTTCCGATCTCGTGTACACCAGCMGCCGCGGTAAT |
| D0 ST-3 | AATGATACGGCGACCACCGAGATCTACACTCTTTCCCTACACGACGCTCTTCCGATCTCGTGTACACCAGCMGCCGCGGTAAT |
| D0 ST-4 | AATGATACGGCGACCACCGAGATCTACACTCTTTCCCTACACGACGCTCTTCCGATCTCGTGTACACCAGCMGCCGCGGTAAT |
| D0 ST-5 | AATGATACGGCGACCACCGAGATCTACACTCTTTCCCTACACGACGCTCTTCCGATCTCGTGTACACCAGCMGCCGCGGTAAT |
| D0 ST-6 | AATGATACGGCGACCACCGAGATCTACACTCTTTCCCTACACGACGCTCTTCCGATCTCGTGTACACCAGCMGCCGCGGTAAT |
| D0 ST-7 | AATGATACGGCGACCACCGAGATCTACACTCTTTCCCTACACGACGCTCTTCCGATCTCGTGTACACCAGCMGCCGCGGTAAT |
| D0 ST-8 | AATGATACGGCGACCACCGAGATCTACACTCTTTCCCTACACGACGCTCTTCCGATCTCGTGTACACCAGCMGCCGCGGTAAT |
| D0 ST-9 | AATGATACGGCGACCACCGAGATCTACACTCTTTCCCTACACGACGCTCTTCCGATCTCGTGTACACCAGCMGCCGCGGTAAT |
| D3 ST-1 | AATGATACGGCGACCACCGAGATCTACACTCTTTCCCTACACGACGCTCTTCCGATCTCTAGCTACCAGCMGCCGCGGTAAT |
| D3 ST-2 | AATGATACGGCGACCACCGAGATCTACACTCTTTCCCTACACGACGCTCTTCCGATCTCTAGCTACCAGCMGCCGCGGTAAT |
| D3 ST-3 | AATGATACGGCGACCACCGAGATCTACACTCTTTCCCTACACGACGCTCTTCCGATCTCTAGCTACCAGCMGCCGCGGTAAT |
| D3 ST-4 | AATGATACGGCGACCACCGAGATCTACACTCTTTCCCTACACGACGCTCTTCCGATCTCTAGCTACCAGCMGCCGCGGTAAT |
| D3 ST-5 | AATGATACGGCGACCACCGAGATCTACACTCTTTCCCTACACGACGCTCTTCCGATCTCTAGCTACCAGCMGCCGCGGTAAT |
| D3 ST-6 | AATGATACGGCGACCACCGAGATCTACACTCTTTCCCTACACGACGCTCTTCCGATCTCTAGCTACCAGCMGCCGCGGTAAT |
| D3 ST-7 | AATGATACGGCGACCACCGAGATCTACACTCTTTCCCTACACGACGCTCTTCCGATCTCTAGCTACCAGCMGCCGCGGTAAT |
| D3 ST-8 | AATGATACGGCGACCACCGAGATCTACACTCTTTCCCTACACGACGCTCTTCCGATCTCTAGCTACCAGCMGCCGCGGTAAT |
| D3 ST-9 | AATGATACGGCGACCACCGAGATCTACACTCTTTCCCTACACGACGCTCTTCCGATCTCTAGCTACCAGCMGCCGCGGTAAT |
| Samples | Reverse primers |
| D0 ST-1 | CAAGCAGAAGACGGCATACGAGATCGGCATTCCTGCTGAACCGCTCTTCCGATCTTGGTCACCCGTCAATTYYTTTRAGTTT |
| D0 ST-2 | CAAGCAGAAGACGGCATACGAGATCGGCATTCCTGCTGAACCGCTCTTCCGATCTCACTGTCCGTCAATTYYTTTRAGTTT |
| D0 ST-3 | CAAGCAGAAGACGGCATACGAGATCGGCATTCCTGCTGAACCGCTCTTCCGATCTATTGGCCCGTCAATTYYTTTRAGTTT |
| D0 ST-4 | CAAGCAGAAGACGGCATACGAGATCGGCATTCCTGCTGAACCGCTCTTCCGATCTGATCTGCCGTCAATTYYTTTRAGTTT |
| D0 ST-5 | CAAGCAGAAGACGGCATACGAGATCGGCATTCCTGCTGAACCGCTCTTCCGATCTTCAAGCCGTCAATTYYTTTRAGTTT |
| D0 ST-6 | CAAGCAGAAGACGGCATACGAGATCGGCATTCCTGCTGAACCGCTCTTCCGATCTCTGATCCGTCAATTYYTTTRAGTTT |
| D0 ST-7 | CAAGCAGAAGACGGCATACGAGATCGGCATTCCTGCTGAACCGCTCTTCCGATCTAAGCCCGTCAATTYYTTTRAGTTT |
| D0 ST-8 | CAAGCAGAAGACGGCATACGAGATCGGCATTCCTGCTGAACCGCTCTTCCGATCTGTAGCCCGTCAATTYYTTTRAGTTT |
| D0 ST-9 | CAAGCAGAAGACGGCATACGAGATCGGCATTCCTGCTGAACCGCTCTTCCGATCTTACCCCGTCAATTYYTTTRAGTTT |
| D3 ST-1 | CAAGCAGAAGACGGCATACGAGATCGGCATTCCTGCTGAACCGCTCTTCCGATCTTGGTCACCCGTCAATTYYTTTRAGTTT |
| D3 ST-2 | CAAGCAGAAGACGGCATACGAGATCGGCATTCCTGCTGAACCGCTCTTCCGATCTCACTGTCCGTCAATTYYTTTRAGTTT |
| D3 ST-3 | CAAGCAGAAGACGGCATACGAGATCGGCATTCCTGCTGAACCGCTCTTCCGATCTATTGGCCCGTCAATTYYTTTRAGTTT |
| D3 ST-4 | CAAGCAGAAGACGGCATACGAGATCGGCATTCCTGCTGAACCGCTCTTCCGATCTGATCTGCCGTCAATTYYTTTRAGTTT |
| D3 ST-5 | CAAGCAGAAGACGGCATACGAGATCGGCATTCCTGCTGAACCGCTCTTCCGATCTTCAAGCCGTCAATTYYTTTRAGTTT |
| D3 ST-6 | CAAGCAGAAGACGGCATACGAGATCGGCATTCCTGCTGAACCGCTCTTCCGATCTCTGATCCGTCAATTYYTTTRAGTTT |
| D3 ST-7 | CAAGCAGAAGACGGCATACGAGATCGGCATTCCTGCTGAACCGCTCTTCCGATCTAAGCCCGTCAATTYYTTTRAGTTT |
| D3 ST-8 | CAAGCAGAAGACGGCATACGAGATCGGCATTCCTGCTGAACCGCTCTTCCGATCTGTAGCCCGTCAATTYYTTTRAGTTT |
| D3 ST-9 | CAAGCAGAAGACGGCATACGAGATCGGCATTCCTGCTGAACCGCTCTTCCGATCTTACCCCGTCAATTYYTTTRAGTTT |
